# Interferon-Induced Protein 44 and Interferon-Induced Protein 44-Like Restrict Replication of Respiratory Syncytial Virus

**DOI:** 10.1128/JVI.00297-20

**Published:** 2020-08-31

**Authors:** D. C. Busse, D. Habgood-Coote, S. Clare, C. Brandt, I. Bassano, M. Kaforou, J. Herberg, M. Levin, J.-F. Eléouët, P. Kellam, J. S. Tregoning

**Affiliations:** a Department of Infectious Disease, Imperial College London, St. Mary's Campus, London, United Kingdom; b Wellcome Trust Sanger Institute, Wellcome Trust Genome Campus, Hinxton, United Kingdom; c Unité De Virologie et Immunologie Moléculaires (UR892), INRA, Université Paris-Saclay, Jouy-en-Josas, France; d Kymab Ltd., Cambridge, United Kingdom; Hudson Institute of Medical Research

**Keywords:** IFI44, innate immunity, intrinsic immunity, RSV, restriction factor

## Abstract

Cellular intrinsic immunity, mediated by the expression of an array of interferon-stimulated antiviral genes, is a vital part of host defense. We have previously used a bioinformatic screen to identify two interferon-stimulated genes (ISG) with poorly characterized function, interferon-induced protein 44 (IFI44) and interferon-induced protein 44-like (IFI44L), as potentially being important in respiratory syncytial virus (RSV) infection. Using overexpression systems, CRISPR-Cas9-mediated knockout, and a knockout mouse model, we investigated the antiviral capability of these genes in the control of RSV replication. Overexpression of IFI44 or IFI44L was sufficient to restrict RSV infection at an early time postinfection. Knocking out these genes in mammalian airway epithelial cells increased levels of infection. Both genes express antiproliferative factors that have no effect on RSV attachment but reduce RSV replication in a minigenome assay. The loss of *Ifi44* was associated with a more severe infection phenotype in a mouse model of infection. These studies demonstrate a function for IFI44 and IFI44L in controlling RSV infection.

**IMPORTANCE** RSV infects all children under 2 years of age, but only a subset of children get severe disease. We hypothesize that susceptibility to severe RSV necessitating hospitalization in children without predefined risk factors is, in part, mediated at the antiviral gene level. However, there is a large array of antiviral genes, particularly in the ISG family, the mechanism of which is poorly understood. Having previously identified IFI44 and IFI44L as possible genes of interest in a bioinformatic screen, we dissected the function of these two genes in the control of RSV. Through a range of overexpression and knockout studies, we show that the genes are antiviral and antiproliferative. This study is important because IFI44 and IFI44L are upregulated after a wide range of viral infections, and IFI44L can serve as a diagnostic biomarker of viral infection.

## Abstract (precis)

RSV infects all children under 2 years of age, but only a subset of children get severe disease. We hypothesize that susceptibility to severe RSV necessitating hospitalization in children without predefined risk factors is, in part, mediated at the antiviral gene level. However, there is a large array of antiviral genes, particularly in the ISG family, the mechanism of which is poorly understood. Having previously identified IFI44 and IFI44L as possible genes of interest in a bioinformatic screen, we dissected the function of these two genes in the control of RSV. Through a range of overexpression and knockout studies, we show that the genes are antiviral and antiproliferative. This study is important because IFI44 and IFI44L are upregulated after a wide range of viral infections, and IFI44L can serve as a diagnostic biomarker of viral infection.

## INTRODUCTION

Respiratory syncytial virus (RSV) is a major global cause of morbidity in young children and the elderly, representing a significant burden on health care infrastructure (1). The majority of RSV infections in susceptible populations are self-limiting; however, some 2% of infected infants develop a severe infection and require hospitalization. The risk factors behind this development of severe RSV disease have yet to be fully elucidated, and some 75% of hospitalized infants present with no known risk factor (2, 3). This suggests that there is a genetic element to susceptibility to symptomatic infection, and since interferon (IFN)-stimulated genes (ISGs) are vital in early viral control, they are a likely candidate. A number of ISGs have been demonstrated to inhibit RSV, including IFITM proteins (4, 5), TDRD7 (6), and 2′-5′ oligoadenylate synthetase (7).

A vital component of the innate host response to viral infection is the intracellular amplification of an array of antiviral proteins in response to type I IFN. The majority of these inducible proteins, encoded by ISGs, have no defined function and have only been poorly characterized in terms of their antiviral tropism. Understanding how these genes reduce viral infection gives insight into the viral life cycle and may open up novel therapeutic routes. We have previously performed a bioinformatic screen of ISG expressed after RSV infection, which prioritized ISGs of interest for further study (8).

Two ISGs of interest identified in our previous bioinformatic screen are *IFI44* and *IFI44L*, which are found adjacently on chromosome one. *IFI44L* is a gene of 26 kb, larger than the 14 kb of *IFI44*, but both genes encode similar-sized proteins translated from a transcript produced from nine exons. IFI44 is made up of 444 amino acids, whereas IFI44L has 452 residues; the two proteins share 45% amino acid identity. *IFI44*, previously known as *MTAP44*, was first identified in the context of hepatitis C virus infection (9, 10). Overexpression of IFI44 has been shown to restrict Bunyamwera virus (11) and HIV-1 (12) infection *in vitro*. IFI44 was initially described as a cytoplasmic protein; however, two studies have observed that low levels of IFI44 can be found in the nucleus (12, 13). Hallen et al. reported that the overexpression of IFI44 was able to reduce the proliferation of two melanoma cell lines independently of IFN-I (13). The antiproliferative mechanism of IFI44 remains unexplored.

Even less is known about the tropism and function of IFI44L. IFI44L has been shown to have a moderate impact on hepatitis C virus infection (14). Interestingly, *IFI44L* expression has also been associated with several autoimmune disorders (15–17), cancer (18, 19), and humoral responses to vaccination (20). These seemingly disparate contexts suggest that *IFI44L* is a biomarker of IFN responses independent of the type of stimulus. Interestingly, IFI44L expression is sufficient to distinguish viral from bacterial infection (21). Like IFI44, IFI44L has antiproliferative activity, associated with increased activation of Met/Src signaling (18).

Using both overexpressing and knockout (KO) cell lines, we demonstrated that IFI44 and IFI44L are antiproliferative factors that can independently restrict RSV infection. We report that this ability to restrict infection involves the reduction of viral genome transcription or replication but was not dependent upon a predicted guanosine-5′-triphosphate (GTP)-binding region present in either protein. We demonstrate, for the first time, that the loss of IFI44 expression in a mouse model of infection is associated with more severe RSV disease.

## RESULTS

### *IFI44* and *IFI44L* are upregulated early in response to IFN-I and RSV.

**FIG 1:**
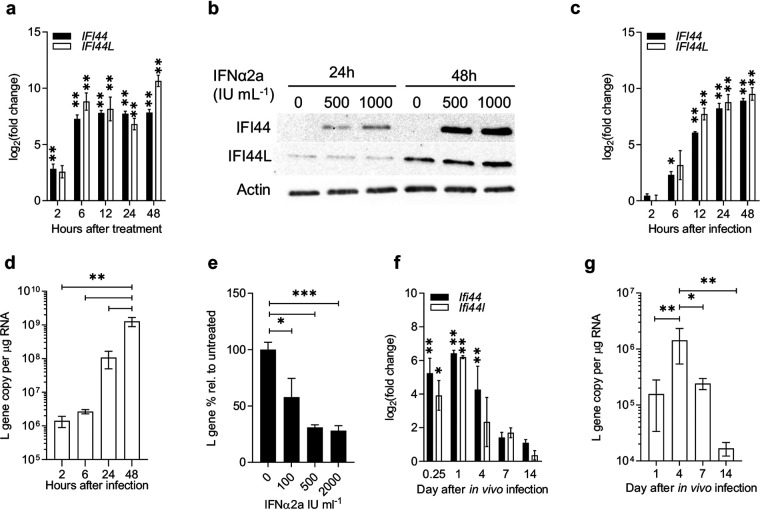
*IFI44* and *IFI44L* are IFN-I-responsive genes upregulated during RSV infection. (a) *IFI44* and *IFI44L* mRNA expression in A549 cells treated with 500 IU ml^−1^ IFN-α2a. (b) IFI44 and IFI44L protein levels in A549 cells treated with 0 to 1,000 IU ml^−1^ IFN-α2a for 24 or 48 h. (c and d) *IFI44* and *IFI44L* mRNA expression (c) and RSV L gene copies (d) in A549 cells infected with RSV A2 (MOI of 0.1) for 2 to 24 h. *N* ≥ 3. (e) A549 cells treated with IFN-α2a for 16 h prior to infection with RSV A2 (MOI of 0.1) for 24 h. Viral RNA is shown relative to untreated controls. *N* = 3. (f) Eight- to 10-week-old BALB/c mice were infected intranasally with 2 × 10^5^ PFU RSV A2. Shown is the change in expression of *Ifi44* and *Ifi44l* mRNA in infected mice relative to mice given PBS intranasally at the time of infection. (g) Total L gene copy number per microgram of RNA from whole-lung tissue. *N* ≥ 4 animals per group at each time point. Data are presented as the means ± standard errors of the means (SEM). Significance relative to untreated controls (a, c, and e), PBS-treated groups (f), or indicated groups (d and g) was assessed by analysis of variance (ANOVA). *, *P* < 0.05; **, *P* < 0.01; ***, *P* < 0.001.

We have previously identified a number of ISGs that are consistently upregulated in response to RSV (8), of which IFI44 and IFI44L featured prominently. We focused on these two genes, as relatively little was known of their phenotypes. Human lung epithelial A549 cells treated with recombinant IFN-α2a robustly upregulated expression of *IFI44* and *IFI44L* mRNA within 2 and 6 h, respectively (*P* < 0.01) (Fig. 1a). Expression remained upregulated for at least 48 h following IFN treatment. IFI44 protein was increased following 24 to 48 h of IFN-α2a stimulation (Fig. 1b) and was undetectable in unstimulated cells. IFI44L protein was detectable in unstimulated cells, and it did not appear to be induced by IFN treatment relative to the level for the control untreated cells (Fig. 1c).

Cells were infected with RSV A2, and mRNA induction following RSV infection was slower than that for IFN, with *IFI44* and *IFI44L* upregulated from 6 h (*P* < 0.05) (Fig. 1b). IFI44 and IFI44L protein levels were not measured after RSV infection. Viral RNA was detectable at 24 h after infection (Fig. 1d). When cells were pretreated with IFN-α2a before infection, there was a significant reduction in viral RNA levels (Fig. 1e).

*In vivo*, the expression of both *Ifi44* and *Ifi44l* RNA was significantly upregulated rapidly after intranasal infection of BALB/c mice, detectable from 6 h (*P* < 0.05) (Fig. 1f). Gene expression peaked after 24 h and returned toward baseline levels by day 14, in parallel with levels of viral RNA that also reduced as the infection was cleared (Fig. 1g).

### Overexpression of IFI44 or IFI44L restricts RSV infection.

**FIG 2:**
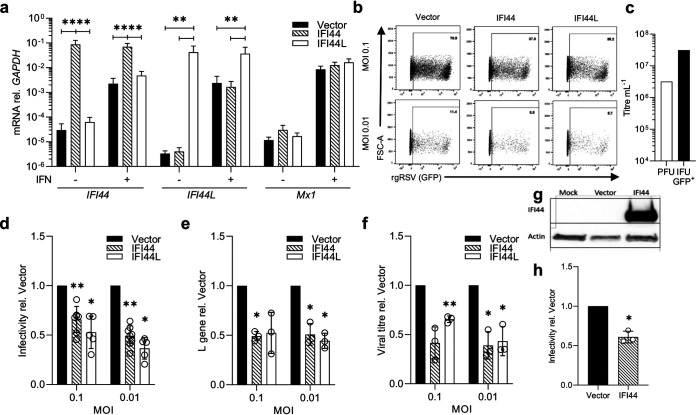
Overexpression of IFI44 or IFI44L reduces RSV infection. (a) *IFI44*, *IFI44L*, and *Mx1* mRNA levels relative to that of *GAPDH* in stably transduced overexpressing monoclonal A549 cell lines. Cells were cultured in normal growth medium or medium supplemented with 500 IU ml^−1^ IFN-α2a for 24 h. (b) Stably transduced cell lines were infected with rgRSV (MOI of 0.1 or 0.01) and infectivity assessed by flow cytometry after 24 h. Representative experiment showing GFP^+^ population of single RFP^+^ cells. (c) Comparison of PFU and GFP^+^ IFU. (d) RSV infection of stably transduced cell lines relative to the vector control. *N* ≥ 5. (e) RSV L gene 24 h postinfection with wild-type RSV A2 relative to vector control. (f) Viral titer (WT RSV A2) relative to vector control in cell supernatant at 48 h postinfection by plaque assay. (g) A549 cells were transduced with either FLUC (vector) or IFI44 lentivirus, and the expression of IFI44 was detected by Western blotting after 48 h. (h) Transduced A549 cells were infected with rgRSV (MOI of 0.8) after 24 h, and the infectivity of transduced (RFP^+^) cells was assessed 24 h after infection. *N* = 3. Individual points represent the results from an independent experiment. Bars show the means ± SEM. Asterisks represent significance relative to values for cells transduced with empty vector, assessed by ANOVA (a) or ratio-paired *t* test (c to e). Analysis was done prior to data transformation. *, *P* < 0.05; **, *P* < 0.01; ***, *P* < 0.001; ****, *P* < 0.0001.

In a previous screen to identify ISGs that impact RSV infection, McDonald et al. showed that transient overexpression of human IFI44 or IFI44L by lentiviral transduction reduced the percentage of RSV-infected cells (8). Here, we generated stably transduced clonal cell lines by lentiviral transduction, followed by fluorescence-activated cell sorting (FACS) selection. Following expansion, clonal populations were selected that expressed TagRFP after 3 weeks in culture following sorting. Individual cell lines were then selected based on detectable expression of either *IFI44* or *IFI44L* by quantitative PCR (qPCR) (Fig. 2a). *MX1* expression was assessed in each stable cell line to confirm the specificity of overexpression to either *IFI44* or *IFI44L*. There were no significant differences in expression of *MX1* in either cell line following IFN-I stimulation, suggesting that neither IFI44 nor IFI44L is a regulator of the IFN response.

To monitor the impact on infection, cells were infected at a multiplicity of infection (MOI) of 0.1 or 0.01 for recombinant green fluorescent protein (GFP)-expressing RSV (rgRSV). The MOI was based on plaque assay titer. The percentage of red fluorescent protein-positive (RFP^+^) single cells that were GFP^+^ was quantified after 24 h (Fig. 2b). The percentage of cells infected as measured by GFP^+^ signal was greater than that expected based on the MOI. Therefore, we compared the number of PFU with the number of inclusion-forming units (IFU; GFP count) for the same vial of virus and saw the number of IFU was 10-fold higher (Fig. 2c). For consistency, we continued the use of MOI based on the number of PFU, because that allowed the comparison of wild-type and fluorescent virus.

After 24 h of rgRSV infection, cell lines expressing either IFI44 or IFI44L showed a significant reduction in the percentage of infected cells relative to cells stably transduced with empty vector (*P* < 0.05) (Fig. 2d). To confirm the impact on RSV infection, we examined the impact on wild-type RSV A2 infection in these stable cell lines. We observed a significant reduction in viral RNA 24 h after infection (MOI of 0.01) (*P* < 0.05) (Fig. 2e). To observe the impact on virus progeny production, we measured the viral titer 48 h after infection. The recoverable titer of RSV A2 virus was significantly reduced in cells expressing either IFI44 or IFI44L (*P* < 0.05) (Fig. 2f).

To demonstrate that the impact of IFI44 expression is not a result of clonal differences, we transduced polyclonal A549 cells with lentivirus expressing either Firefly luciferase (FLUC) or IFI44 and subsequently infected the cells with rgRSV after 24 h. Expression of IFI44 protein was confirmed (Fig. 2g) and significant restriction in rgRSV infection (*P* < 0.05) (Fig. 2h) was observed, similar to that seen in the stably transduced cell line. Overall, these data suggest that both IFI44 and IFI44L can restrict RSV infection.

### Knockout of *IFI44* results in elevated RSV infection *in vitro*.

**FIG 3:**
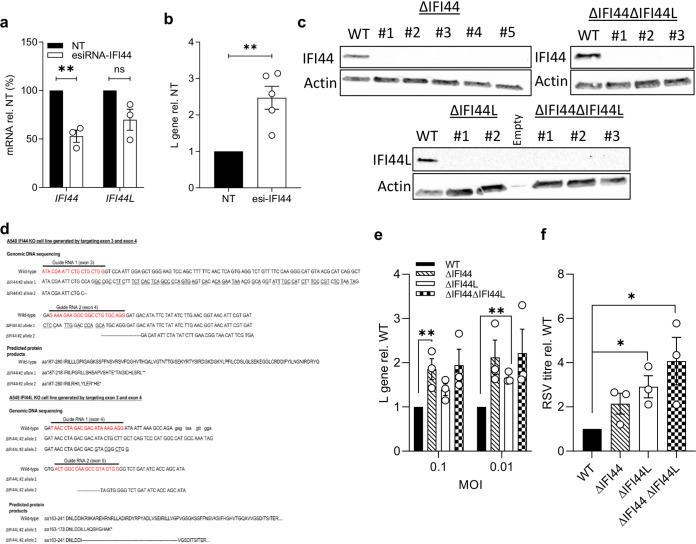
Loss of IFI44 expression enhances RSV infection *in vitro*. (a) *IFI44* and *IFI44L* mRNA expression after transfection with 50 nM esiRNA-IFI44 or a nontargeting esiRNA control (NT). Twenty-four hours after transfection, cells were treated with 500 IU ml^−1^ IFN-α2a for 16 h prior to infection with RSV A2 (MOI of 0.1) for 48 h. *N* = 3. (b) L gene copy number assessed 48 h after infection and shown relative to the NT control. *N* = 5. (c) A549 monoclonal knockout cell lines were generated through CRISPR-Cas9 gene editing. IFI44 or IFI44L protein in wild-type cells treated with IFN-α2a or monoclonal CRISPR-Cas9-edited A549 cells. (d) Sequence and predicted protein of CRISPR-generated lines. (e) Cells were treated with 500 IU ml^−1^ IFN-α2a 16 h prior to infection with RSV A2 (MOI of 0.1 or 0.01) for 24 h. RSV L gene copy number is shown relative to the WT control. *N* = 3. (f) Knockout cell lines were pretreated with IFN-α2a as described for panel c and subsequently infected with RSV A2 (MOI of 0.1) for 48 h. Viral titer in cell supernatant is relative to that of the WT control. *N* = 3. Points represent a single independent experiment with a bar at the means ± SEM. Significance relative to the WT or NT control by ratio-paired *t* test. Analysis was done prior to data transformation. *, *P* < 0.05; **, *P* < 0.01. ns, not significant.

To further examine the role of IFI44 or IFI44L in RSV infection, we used a pool of endoribonuclease-prepared short interfering RNAs (esiRNAs) targeting *IFI44* to knock down expression. These esiRNAs reduced levels of *IFI44* mRNA by only 48% (*P* < 0.01) and also reduced *IFI44L* mRNA levels by 30%, although this was not statistically significant (Fig. 3a). However, esiRNA knockdown was sufficient to cause a more than 2-fold increase in the levels of viral RNA in esiRNA-IFI44-transfected A549 cells infected with RSV A2 (MOI of 0.1) relative to cells transfected with a nontargeting control (*P* < 0.01) (Fig. 3b).

Due to the low efficiency of the knockdown and potential unintended impact of siRNA on *IFI44L* expression, we developed CRISPR-Cas9-edited IFI44- and IFI44L-deficient cell lines; this also enabled us to investigate the impact of double knockouts. A549 cells were transfected with two guide RNA (gRNA) constructs encoded in *cis* with the Streptococcus pyogenes Cas9 enzyme and a GFP marker. gRNA sequences targeting regions in exons 4 and 5 of *IFI44* and exons 3 and 4 of *IFI44L* were used. Following transfection, the top 2% of GFP^+^ cells were sorted as single cells. Gene editing was confirmed in clonal populations through PCR of the targeted region (data not shown) and the loss of either IFI44 or IFI44L protein expression after treatment with IFN-α (Fig. 3c). Five IFI44-deficient (ΔIFI44), two IFI44L-deficient (ΔIFI44L), and three clonal populations deficient in both IFI44 and IFI44L (ΔIFI44ΔIFI44L) were isolated. The selection of clones was based on sequencing (Fig. 3d). The ΔIFI44 clone selected for further study had mutations resulting in a frameshift and stop codon formation in each detectable allele. The ΔIFI44L clone selected had a large deletion in one allele and disruption of an exon-intron boundary.

The selected clones were treated with 500 IU ml^−1^ IFN-α2α and subsequently infected with RSV A2 for 24 h. Cells were pretreated with IFN because basal protein expression was low; therefore, we would not expect to see an impact of gene knockout. There were significantly increased levels of viral RNA in the ΔIFI44 clone at an MOI of 0.1 (Fig. 3e) (*P* < 0.01), although we noted a nonsignificant increase in infection in the ΔIFI44ΔIFI44L cells (*P* = 0.079). The loss of IFI44L expression was only associated with a significant increase in viral RNA at an MOI of 0.01 (*P* < 0.01). Clones were infected with RSV A2 (MOI of 0.1) for 48 h and viral titer assessed in the culture medium; there was a >2-fold increase in viral titer (Fig. 3f) in each clone. Knockout of both *IFI44* and *IFI44L* resulted in a 4-fold increase in viral titer (Fig. 3f) (*P* < 0.05). A slight difference between the two readouts, RNA and viral titer, was observed.

### IFI44 and IFI44L reduce cellular proliferation.

**FIG 4:**
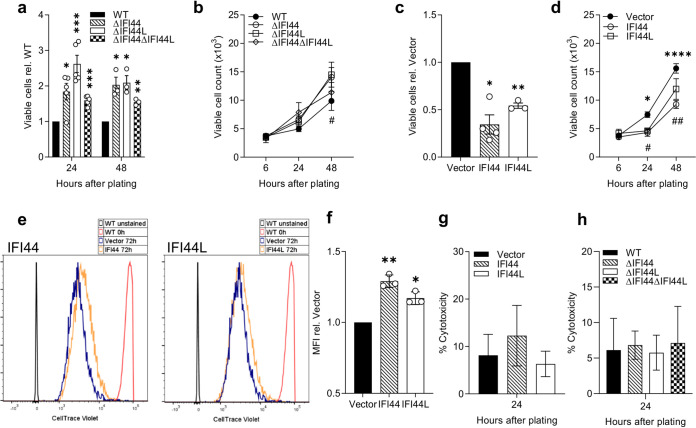
IFI44 and IFI44L are antiproliferative. A549 monoclonal knockout cell lines were seeded at equal densities and viable cell numbers quantified after 6 to 48 h using a colorimetric metabolic activity assay (a) or by trypan blue exclusion (*#*, *P* < 0.05 between ΔIFI44L and vector). (b) A549 stably transduced monoclonal cell lines expressing IFI44 or IFI44L or transduced with empty vector were seeded at equal densities and viable cell numbers quantified 24 h later by colorimetric metabolic activity assay (c) or after 6 to 48 h by trypan blue exclusion (d) (*, *P* < 0.05; ****, *P* < 0.0001 between IFI44 and vector; #, *P* < 0.05; ##, *P* < 0.01 between IFI44L and vector). *N* ≥ 3. (e) Representative histograms of stably transduced A549 cell lines treated with CellTrace Violet and allowed to proliferate for 72 h before analysis by flow cytometry. WT cells were stained or treated with vehicle only and immediately fixed prior to analysis for positive (red) and negative (gray) controls. (f) Mean fluorescence intensity (MFI) of cell trace violet quantified relative to vector control over three independent repeats. (g and h) Cytotoxicity was assessed by lactate dehydrogenase release assay 24 to 48 h after plating in overexpression (g) or knockout (h) cells. *N* = 3. Significance compared to WT or vector controls was assessed by ratio-paired *t* test prior to data transformation. Points represent a single independent experiment with a bar at the means ± SEM. *, *P* < 0.05; **, *P* < 0.01; ***, *P* < 0.001; ****, *P* < 0.0001 in panels a, c, and f between the indicated bar and vector.

Previous studies have described an effect of IFI44 or IFI44L on cell proliferation (13, 18). We investigated the impact of these factors on proliferation by assaying the growth of selected knockout clones. Knocking out either or both genes was associated with increased proliferation according to a colorimetric assay measuring cellular metabolic activity (*P* < 0.05) (Fig. 4a). When viable cell numbers were quantified manually by Trypan blue exclusion, only the *IFI44* KO was associated with a significant increase in cell number (*P* < 0.05) (Fig. 4b). Overexpression of either gene led to a significant reduction in proliferation after 24 h, as quantified by either method (*P* < 0.05) (Fig. 4c and d). Cells perfused with CellTrace Violet dye were allowed to proliferate for 72 h prior to analysis by flow cytometry to assess cell division. We noted that both IFI44 and IFI44L stably transduced cell lines had an increased mean fluorescence intensity (*P* < 0.05) (Fig. 4f), suggesting reduced dye dilution and a reduced rate of cell division. The overexpression of IFI44 or IFI44L was not associated with a significant increase in cytotoxicity (Fig. 4g), although there was a nonsignficant increase in the IFI44 line. Likewise, there was no difference in cytotoxicity of the knockout cell lines (Fig. 4h), further suggesting that the observed reduction in viable cell number and proliferation is not a result of increased cell death.

### IFI44 reduces RSV polymerase activity.

**FIG 5:**
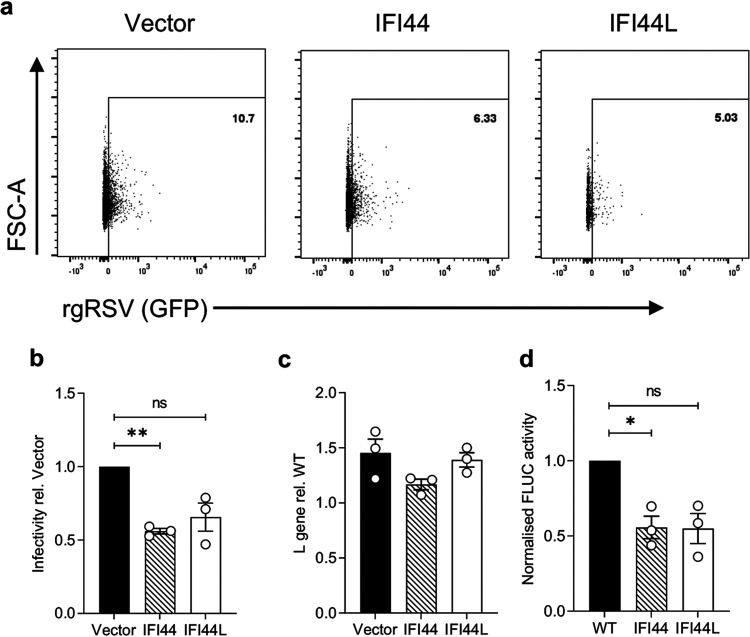
IFI44 and IFI44L reduce RSV polymerase activity but not entry. (a) Stably transduced A549 cells were infected with rgRSV (MOI of 0.1) for only 8 h and infectivity assessed by measuring the percentage of RFP^+^ single cells that were GFP^+^. Representative dot plot from a single independent experiment. Population shown was gated for single RFP^+^ cells. (b) Quantification of rgRSV infection relative to that of the vector-transduced control as described for panel a. *N* = 3. (c) Stably transduced A549 cell lines were incubated with RSV A2 (MOI of 2) for 1 h at 4°C and then harvested for analysis of RSV L gene copy number. *N* = 3. (d) Stably transduced 293T cell lines were transfected with pSV-β-Gal, pCAGGS-T7, the pGEM3-Gaussia/Firefly minigenome, and plasmids encoding RSV M2-1, P, L, and N. Twenty-four hours later, FLUC activity was assessed and normalized to the negative control and β-galactosidase expression levels. Normalized FLUC activity is shown relative to that of polyclonal parental 293T cells (WT). *N* = 3. Significance to vector-transduced or WT cells was assessed by ratio-paired *t* test prior to data transformation. Points represent a single independent experiment with a bar at the means ± SEM *, *P* < 0.05; **, *P* < 0.01. ns, not significant.

To investigate where in the viral life cycle IFI44 and IFI44L have an impact, we analyzed infection at an acute time point (8 h) where virus-positive cells should only be newly infected cells and not the result of cell-cell virus spread. IFI44 or IFI44L expression reduced the percentage of infected cells by 44% (*P* < 0.01) and 34% (*P* = 0.11), respectively (Fig. 5a and b), relative to vector control cells, suggesting both proteins are impacting a stage of the viral life cycle prior to new virion release. Using a cold-bind infection assay (RSV A2; MOI of 2), where the virus can bind the cell surface but is not internalized, we saw no significant difference in levels of viral RNA between cell lines expressing either FLUC (vector), IFI44, or IFI44L (Fig. 5c). To bypass cell entry and to assess whether IFI44 or IFI44L restricts RSV genome replication or transcription, we transfected stably transduced clonal 293T cells with an RSV minigenome system. IFI44 expression reduced minigenome activity by 44% (*P* < 0.05), suggesting reduced RSV polymerase activity (Fig. 5d). The stably transduced 293T line expressing IFI44L also reduced minigenome activity by 45%, although this was not statistically significant (*P* = 0.085).

### Disease severity is altered in an *Ifi44^−/−^/Ifi44L^−/−^* mouse model of RSV infection.

**FIG 6:**
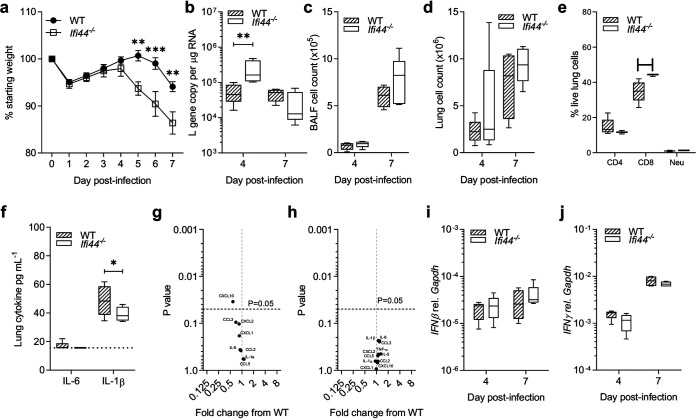
RSV infection severity is enhanced in an *Ifi44^−/−^* mouse model. (a) Wild-type or *Ifi44^−/−^* C57BL/6N mice were infected intranasally with 1 × 10^5^ PFU RSV A2. Weight loss was monitored for 7 days. (b) Viral load assessed by RSV L gene qPCR. (c and d) BALF (c) and lung (d) cell counts. (e) Lung cell types by flow cytometry at d7. (f) IL-6 and IL-1β levels at day 4 postinfection as measured by ELISA. (g and h) Volcano plot of inflammatory cytokines at day 4 (g) and day 7 (h) by multiplex ELISA. (i and j) *Ifnβ* (i) and *Ifnγ* (j) mRNA level relative to that of *Gapdh* (2^−Δ^*^CT^*). Box plots show a line at the median and box edges from the 25th to 75th percentiles, with whiskers from the 5th to 95th percentiles (Prism 8). *N* ≥ 4 animals at each time point. Two independent experiments were performed. Significance was determined by ANOVA (a to e, h, and i) or Student's *t* test (f and g). *, *P* < 0.05; ** *P* < 0.01; ***, *P* < 0.001.

Having demonstrated that IFI44 and IFI44L were able to impact RSV infection *in vitro*, we then investigated the effect of the absence of *Ifi44* and *Ifi44L* in an *in vivo* mouse model of RSV infection. The wild-type C57BL/6N mice used in this study are Ifi44L^−/−^, presumably as a result of gene loss over colony in-breeding, so the comparison was between IFI44^−/−^/IFI44L^−/−^ and IFI44^+/+^/IFI44L^−/−^ (WT) mice. Age-matched *Ifi44^−/−^* and WT mice were infected intranasally with 10^5^ PFU RSV A2 and monitored for weight change over a 7-day infection. Animals were sacrificed at days 4 and 7 after infection. There was greater weight loss in *Ifi44^−/−^* mice than in wild-type controls from days 5 to 7 after infection (*P* < 0.01) (Fig. 6a). Levels of viral RNA in lung tissue were also significantly higher in *Ifi44^−/−^* mice at day 4 (*P* < 0.01) but not at day 7 (Fig. 6b). There was no difference in total cell numbers isolated from the bronchoalveolar lavage fluid or from whole lung tissue (Fig. 6c and d). There was a significantly higher percentage of live CD8^+^ cells in the lungs of knockout mice (Fig. 6e).

Levels of inflammatory cytokines and immunomodulatory factors were analyzed either by enzyme-linked immunosorbent assay (ELISA) or Luminex. IL-1β (*P* < 0.05) (Fig. 6f) and CXCL10 (*P* < 0.05) (Fig. 6g) were both significantly but modestly reduced in the KO animals on day 4 after infection. Most measured analytes (IL-6, CCL3, CXCL2, CXCL1, CCL2, IL-5, IL-1α, and CCL5) were not significantly different between the WT and *Ifi44^−/−^* groups (Fig. 6f to h). There was no difference in any measured cytokine on day 7 after infection (Fig. 6h). To determine whether the lack of IFI44 modulated IFN responses to RSV infection, both *Ifn-β* and *Ifn-γ* expression was assessed by qPCR. Both wild-type and *Ifi44^−/−^* groups demonstrated similar levels of *Ifn-β* (Fig. 6i) and *Ifn-γ* (Fig. 6j) mRNA.

### IFI44 and IFI44L expression in human infants with severe RSV infection.

**FIG 7:**
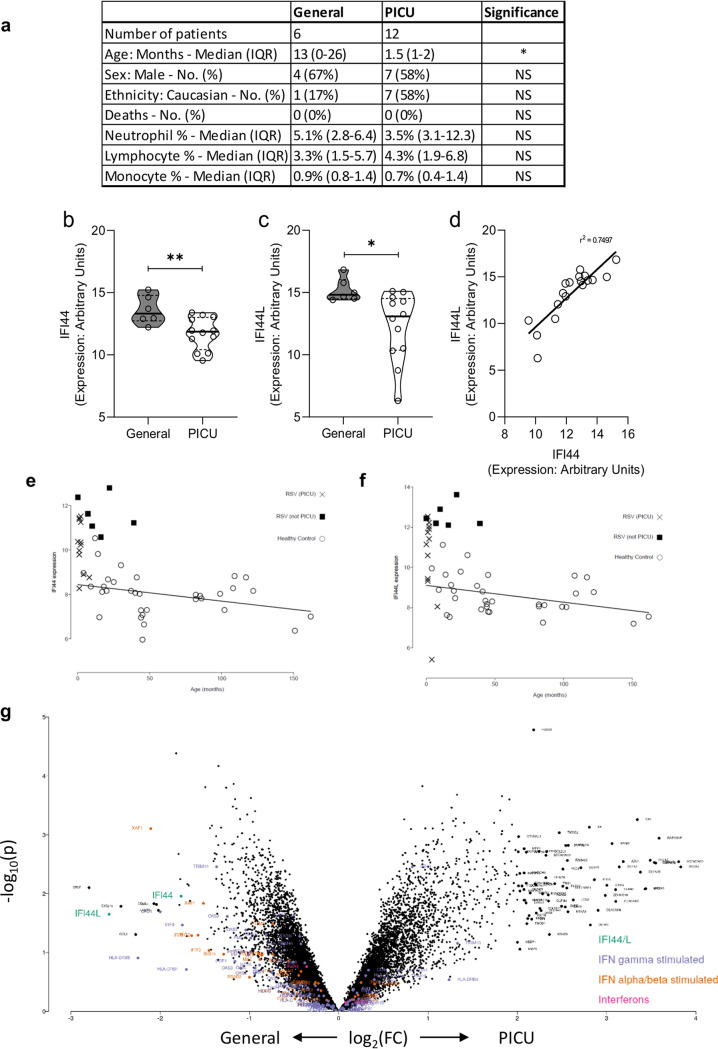
IFI44 and IFI44L mRNA levels are reduced in the blood of infants with severe RSV infection. (a) Demographic and clinical features of patient groups. Patients were febrile children with immunofluorescence-confirmed RSV infection. Patients with suspected or confirmed bacterial coinfection were excluded (*n* = 4). IQR, interquartile range. (b and c) IFI44 (b) or IFI44L (c) RNA expression levels and measured blood PBMCs by microarray in patients admitted to a general ward with mild RSV illness or admitted to a pediatric intensive care unit (PICU) at the same hospital. *, *P* < 0.05; **, *P* < 0.01. Significance was determined by unpaired *t* test. (d) Pearson correlation analysis of expression of IFI44 and IFI44L. *P* < 0.001. (e and f) Baseline expression of IFI44 (e) and IFI44L (f) in healthy children of different ages. (g) Volcano plot of fold change in gene expression by microarray between general hospital and intensive care admission.

Having seen an effect *in vitro* and in mouse models, we examined gene expression levels of both *IFI44* and *IFI44L* in human RSV infection. We used data previously generated by microarray on RNA extracted from whole-blood-derived peripheral blood mononuclear cells (PBMCs) collected from children with confirmed RSV infection. We compared children who required pediatric intensive care unit (PICU) admission with those who were admitted to a general hospital ward (General) (Fig. 7a). When investigated as individual genes in the data set, the expression of both IFI44 (*P* = 0.0082) (Fig. 7b) and IFI44L (*P* = 0.0248) (Fig. 7c) was significantly lower in those patients admitted to the PICU. IFI44 expression correlated with IFI44L expression across both moderate and severe RSV patients (*P* < 0.001, *r*^2^ = 0.74) (Fig. 7d). There was a significant difference in median age between the PICU (1.5 months) and General (13 months) cohorts, but basal expression of IFI44 (Fig. 7e) and IFI44L (Fig. 7f) declines with age in healthy controls, suggesting that this is not a factor. It should be noted that, when investigated in the context of global gene expression data, the differences between general hospital and intensive care admission were not significant, although IFI44L did have a greater than 2-log fold change (Fig. 7g).

## DISCUSSION

The data presented here explore how the ISGs IFI44 and IFI44L modulate viral infection. We demonstrate that IFI44 and IFI44L restrict RSV infection and reduce RSV genome replication or transcription. We also show, for the first time, that RSV infection is enhanced in an *Ifi44^−/−^/Ifi44L^−/−^* knockout mouse model. Infectivity was reduced by IFI44 expression at just 8 h after infection, suggesting restriction of infection occurs before the exit of new virions. Virus attachment was unaffected by either IFI44 or IFI44L expression. This was expected, as these proteins both are predicted to be internally expressed (12, 13). Using an RSV minigenome assay, we found that IFI44 expression significantly reduced RSV polymerase activity. However, we cannot say whether this is an impact specifically on the replication or transcription of the viral genome. We also observed that both IFI44 proteins decreased the rate of cellular proliferation. Reduced proliferation is a common feature of the IFN response, mediated by canonical ISGs such as protein kinase R (PKR) (22) and the IFN-induced tetratricopeptide repeat (IFIT) family (23). Whether the antiproliferative function of the IFI44 proteins is a causative mechanism of their antiviral activity is not clear, because cell cycle arrest may increase the availability of cellular machinery required for replication and virus assembly (24). For example, the RSV matrix protein (M) has previously been shown to induce cell cycle arrest by inducing p53/p21 expression in alveolar epithelial cells, enhancing infection (24, 25).

Our *in vitro* studies have some limitations, primarily the use of clonal cell lines to assay infection and proliferation. We note that the transduction of polyclonal parental A549 cells with IFI44 is capable of restricting RSV infection similarly to the stably transduced cells, and that previous studies have observed similar impacts of IFI44 on proliferation (13, 18) or restriction of RSV (8). However, it is possible that clone-specific differences have some impact on either RSV infection or cell viability, and these data should be interpreted with this limitation in mind. In the knockout cell line, there was a slight difference in the effect on viral RNA and infectious virus recovered, and this may reflect differences in the two assays or a difference in where the ISGs affect replication or packaging.

This is the first study to describe viral infection in *Ifi44^−/−^/Ifi44L^−/−^* mice. *Ifi44^−/−^/Ifi44L^−/−^* mice were markedly more susceptible to RSV infection than WT mice, exhibiting increased weight loss and elevated viral load. The genome sequence of C57BL/6N mice reveals a deletion in *Ifi44l* predicted to ablate expression. We were unable to detect the transcription of this gene in these mice during RSV infection, whereas this was readily detectable in the lungs of RSV-infected BALB/c mice. We observed that *Ifi44^−/−^/Ifi44L^−/−^* mice infected with RSV had higher levels of viral RNA present in their lungs at the peak of infection, along with decreased expression of the proinflammatory factor interleukin-1β (IL-1β). A previous study noted that in adult mice, blockade of IL-1β prior to RSV infection results in elevated viral load (26). Decreased production of this key cytokine along with increased viral replication, due to changes in cellular proliferation and metabolism driven by the loss of IFI44, may go some way to explaining these observations. We saw no change in tumor necrosis factor (TNF) but an increase in CD8 cells; this reflects our recent findings that TNF is associated with early weight loss after RSV infection but not later time points, whereas CD8 cells are associated with reduced food intake leading to weight loss (27).

Our data showing an antiviral role for IFI44 and IFI44L matches our previous study using a lentivirus screen (8) and a broader screen by another group using the same lentivirus panel (14). However, the data presented here are somewhat at odds with recently published studies investigating the impact of IFI44 (28) and IFI44L (29) on virus *in vitro*. In the published studies reducing IFI44 or IFI44L *in vitro*, using siRNA led to increased viral recovery, which was hypothesized to be linked to decreased ISG expression. We did not see an effect of IFI44 or IFI44L overexpression on the expression of the ISG Mx1. One possible difference between study designs is the role of cell proliferation. We observed that IFI44 and IFI44L had a significant antiproliferative effect, and to normalize, we counted cell numbers in parallel wells prior to infections and altered the viral inoculum to ensure equivalent MOIs were used. The parallels between the *in vivo* and *in vitro* phenotype give us confidence that the genes have an antiviral function.

One question of interest concerns the global redundancy of ISGs in the control of infection. Using knockout mouse models, an increase in RSV disease severity has been seen for a variety of individual antiviral ISGs, such as Ifitm3 (30), Ifitm1 (5), and Irf7 (8). It is curious that in these *in vitro* and mouse models, single-gene loss can result in the loss of viral control, when the host network of ISGs consists of potentially over 1,000 genes. While some associations between individual ISGs and disease severity have been observed in humans, for example, IFITM3 (31, 32), more often primary immunodeficiencies caused by mutations in the interferon sensing and signaling pathways, such as STAT1 and TLR3, display incomplete penetrance and only susceptibilities to specific pathogens (33). The use of large volumes relative to lung size and high doses of virus to ensure infection in the mouse model may stress the system so that the role of individual genes becomes more apparent. In the infant data set, we did not see a significant difference in IFI44 and IFI44L expression at the whole transcriptome level. This suggests that these genes are not the sole determinants of outcome after RSV infection and that ISGs work in concert to control infection, targeting different aspects of the viral life cycle, with some factors such as IFITM1 controlling entry (5) and others restricting replication within the cell. One confounding factor is that the children requiring intensive care were younger; however, expression levels of IFI44 and IFI44L in healthy controls were fairly uniform across different-aged children.

Our study demonstrates that IFI44 and IFI44L play a role in the control of RSV infection *in vitro* and in mouse models, reducing the ability of the virus to replicate. Since the proteins are antiproliferative, this may be part of the mechanism. IFI44 and IFI44L have a high degree of homology, and whether they have distinct mechanisms or are redundant is unclear at this time. Understanding how they reduce viral replication may provide future avenues for therapeutic interventions.

## MATERIALS AND METHODS

### Cell culture and viruses.

HEp-2 (from P. Openshaw, Imperial College London), A549 (ATCC CCL-185), and HEK293T/17 (ATCC CRL-11268) cells were maintained in Dulbecco’s modified Eagle medium supplemented with 10%, vol/vol, fetal calf serum, 1%, vol/vol, penicillin-streptomycin, and 1%, vol/vol, l-glutamine. RSV strain A2 (from P. Openshaw, Imperial College London) and rgRSV (34) were passaged in HEp-2 cells before quantification of viral titer by plaque assay. MOI for infection experiments was based upon plaque assay titer. Vesicular stomatitis virus glycoprotein-pseudotyped lentiviral particles were produced by triple transfection in HEK293T/17 cells using Lipofectamine 3000 (ThermoFisher). Lentiviral vectors pTRIP-FLUC-tagRFP, pTRIP-IFI44-tagRFP, and pTRIP-IFI44L-tagRFP were a kind gift from M. Dorner (Imperial College London). To generate mutant proteins, these vectors were altered by using QuikChange XL site-directed mutagenesis (Agilent Technologies) according to the manufacturer’s instructions. Lentivirus was harvested 24 to 52 h after transfection, and the concentration of transducing units (TU) was determined by flow cytometry. For transduction, 5 × 10^4^ cells were seeded into each well of a 24-well plate. After 24 h, cells were transduced with 2 × 10^5^ TU ml^−1^ lentivirus containing supernatant. Stably transduced clonal populations were recovered following fluorescence-activated cell sorting (FACS). RFP expression was monitored over 3 weeks, and the expression of IFI44 or IFI44L was confirmed by quantitative PCR (qPCR) or Western blotting.

### qPCR.

For analysis of *in vitro* samples, cells were lysed in RLT buffer and RNA extracted using a Qiagen RNeasy kit according to the manufacturer’s instructions (Qiagen). RSV viral load *in vivo* was assessed by extracting RNA from frozen lung tissue using TRIzol extraction after disruption in a TissueLyzer (Qiagen). Complementary DNA (cDNA) was reverse transcribed from RNA extracts using GoScript reverse transcriptase with random primers according to the manufacturer’s instructions (Promega). qPCRs were carried out on a Stratagene Mx3005p thermal cycler (Agilent Technologies). RSV viral load was quantified by amplification of the RSV L gene using 900 nM forward primer (5′-GAACTCAGTGTAGGTAGAATGTTTGCA-3′), 300 nM reverse primer (5′- TTCAGCTATCATTTTCTCTGCCAAT-3′), and 100 nM probe (5′-6-carboxyfluorescein-TTTGAACCTGTCTGAACAT-6-carboxytetramethylrhodamine-3′) in TaqMan Universal master mix, no AmpErase UNG (ThermoFisher). Absolute copy number was calculated by comparison to a plasmid standard. mRNA was amplified with SYBRselect master mix (ThermoFisher) according to the manufacturer’s instructions. The following primers were used at a final concentration of 250 nM: hIFI44 (forward, 5′-TGGTACATGTGGCTTTGCTC-3′; reverse, 5′-CCACCGAGATGTCAGAAAGAG-3′), hIFI44L (forward, 5′-AAGTGGATGATTGCAGTGAG-3′; reverse, 5′-CTCAATTGCACCAGTTTCCT-3′), hGAPDH (forward, 5′-GGACCTGACCTGCCGTCTAG-3′; reverse, 5′-TAGCCCAGGATGCCCTTGAG-3′), m*Ifi44* (forward, 5′-AACTGACTGCTCGCAATAATGT-3′; reverse, 5′-GTAACACAGCAATGCCTCTTGT-3′), m*Ifi44l* (forward, 5′-AGTGACAGCCAGATTGACATG-3′; reverse, 5′-CATTGTGGATCCCTGAAGAGAA-3′), and m*Gapdh* (forward, 5′-AGGTCGGTGTGAACGGATTTG-3′; reverse, 5′-TGTAGACCATGTAGTTGAGGTCA-3′). Fold change in target gene in treated samples was calculated using the ΔΔ*C_T_* method (*C_T_*, cycle threshold) and normalized to a reference transcript (35).

### Western blotting.

Cells were lysed using radioimmunoprecipitation assay buffer (Sigma) containing 1× cOmplete Ultra protease inhibitor cocktail (Sigma). Proteins were separated by SDS-PAGE using 4% to 20% precast Mini-Protean TGX gels (Bio-Rad) before transfer onto a nitrocellulose membrane using the Trans-Blot turbo transfer system (Bio-Rad). Membranes were blocked for 1 h at room temperature (RT) with 5% milk in phosphate-buffered saline (PBS) with 0.1% Tween 20. Membranes were probed using the following primary antibodies for 16 h at 4°C: IFI44 (PA5-65370; ThermoFisher), IFI44L (ARP46166; VWR), and β-actin (ab8227; Abcam). Membranes were washed and probed with anti-IgG horseradish peroxidase-conjugated antibodies (Dako) prior to chemiluminescent detection.

### Flow cytometry.

Analysis was performed on a Becton, Dickinson Fortessa LSR using a 561-nm laser and 582/15 band pass filter to detect tag-RFP-positive cells and a 499-nm laser and 530/30 band pass filter to detect GFP-positive cells. Acquisition was set to record 10^4^ events followed by doublet gating and analysis with FlowJo V10.

### RSV plaque assay.

RSV titer in cell-free supernatant was quantified by immunoplaque assay using biotinylated goat anti-RSV polyclonal antibody (Abcam). HEp-2 cells were infected with dilutions of RSV-containing supernatant for 24 h. Cells were fixed in methanol containing 2% hydrogen peroxide for 20 min at RT. Cells were washed with 1% bovine serum albumin (BSA) and PBS prior to addition of anti-RSV antibody for 1 h. Plaques were then visualized by incubating the cells with ExtrAvidin peroxidase followed by 3 amino-ethylcarbazole substrate (Sigma).

### CRISPR knockout generation.

Guide RNA (gRNA) sequences targeting human *IFI44* (gRNA1, CAA TAC GAA TTC T; gRNA2, GAA AGA AGG CGG CCT GTG C) and *IFI44L* (gRNA1, TAA CCT AGA CGA CAT AAA G; gRNA2, GTG ACT GGC CAA GCC GTA G) were synthesized according to Sanjana et al. (36) and cloned into pSpCas9(BB)-2A-GFP (number 48138; Addgene) following BbsI digestion. The insertion of gRNA sequences was validated by sequencing (Eurofins Genomics). A549 cells were transfected and sorted by FACS after 48 h. Clonal knockouts were validated by PCR amplification of the targeted region, agarose gel electrophoresis, Western blotting, and sequencing. Clustal Omega was used for multiple-sequence alignment (37).

### Proliferation assays.

Viable cell numbers were quantified either by Trypan blue exclusion or by the production of formazan product (measured by optical density at 490 nm) 2 h after addition of CellTiter 96 Aqueous One solution assay reagent (Promega) according to the manufacturer’s instructions. Different cell lines were seeded at equal densities and viable cell numbers quantified 6 to 48 h later. Cell division was assessed by staining cells with 5 μM CellTrace Violet reagent (ThermoFisher) and flow cytometric analysis (10^4^ single-cell events, 405-nm laser with a 450/50 band pass filter) after 72 h. Prior to analysis, cells were harvested and fixed for 20 min at RT in 4% paraformaldehyde.

### RSV cold-bind assay.

Stably transduced cell lines were seeded at equal densities 24 h prior to infection. Each cell line was counted prior to infection to ensure inocula were normalized across different cell lines. Cells were equilibrated to 4°C for 30 min before medium was removed and infected with RSV A2 in a minimal volume of serum-free DMEM for 90 min at 4°C. Cells then were washed 3× with ice-cold 1× PBS and lysed in RLT buffer (Qiagen) with 1/100 β-mercaptoethanol (Sigma) for quantification of RSV L gene RNA.

### RSV minigenome assay.

The RSV minigenome and plasmids expressing RSV L, N, P, and M2-1 proteins were described previously (38). pGEM3-Gaussia/Firefly encodes a subgenomic RSV replicon: from the 3′ end, A2 leader sequence (Le), Gaussia luciferase open reading frame (ORF) with an NS1 gene start (GS) and M gene end (GE) sequence, Firefly luciferase ORF with SH GS and GE sequences, and A2 trailer region (Tr). HEK293T/17 cells and stably transduced 293T/17 cell lines were seeded 24 h prior to transfection at 90% confluence in 24-well plates. The cells were transfected using Lipofectamine 3000 with a DNA mixture of 0.25 μg pGEM3-Gaussia/Firefly minigenome, 0.125 μg pCITE-L, 0.25 μg pCITE-P, 0.06 μg pCITE-M2-1, 0.25 μg pCITE-N, 0.12 μg pSV-β-Gal (Promega), and 0.25 μg pCAGGS-T7 (number 65974; Addgene). Negative controls were transfected with the DNA mix with pCITE-L replaced by pcDNA3.1. Cells were lysed in 1× passive lysis buffer (Promega) after 24 h. Firefly luciferase activities were measured in 10 μl of lysate using 50 μl luciferase assay substrate (Promega). To normalize transfection efficiencies, β-galactosidase levels were measured using the β-galactosidase enzyme assay system (Promega). Twenty microliters of lysate was diluted 1:1 in 1× reporter lysis buffer before addition of 40 μl 2× assay buffer. Following incubation at 37°C for 1 h, 150 μl 1 M Na_2_CO_3_ was added and absorbance measured at 420 nm.

### Mouse infection.

Background-, sex-, and age-matched >95% C57BL/6N or BALB/c wild-type and *Ifi44^tm1b^*^(^*^komp^*^)^*^Wtsi^* (*Ifi44^−/−^*) mice (39) (Wellcome Trust Sanger Institute) were supplied with food and water *ad libitum* and monitored daily. Mice were infected intranasally (i.n.) with 1 × 10^5^ to 4 × 10^5^ PFU of RSV A2 in 100 μl under isoflurane anesthesia.

### Enzyme-linked immunosorbent assay.

Bronchoalveolar lavage fluid (BALF) was collected by inflating the lungs with PBS. Supernatant was collected after centrifugation and assayed. For lung homogenate, lung tissue was homogenized through a 100-μm cell strainer (Falcon) and supernatant collected after centrifugation and ammonium-chloride-potassium (ACK) lysis. Cytokines in lung homogenate and BALF were quantified using DuoSet ELISAs according to the manufacturer's instructions (R&D Systems).

### Luminex multiplex ELISA.

Lung homogenate supernatant was subjected to a magnetic Luminex assay using a premixed multianalyte kit: CXCL10, CCL3, CXCL2, CXCL1, IL-5, CCL2, IL-1α, and CCL5 (R&D Systems). Samples and diluted microparticles were combined according to the manufacturer’s instructions and incubated for 2 h at RT (800 rpm). A magnet was applied to the bottom of the plate, and wells were washed 3× for 1 min each time before addition of a biotin antibody cocktail (1 h at RT, 800 rpm). The previous wash was repeated, and Streptavidin-phycoerythrin (PE) was added to each well (30 min at RT, 800 rpm). The wash was repeated and microparticles resuspended in wash buffer for analysis on a Bio-Plex 100 Luminex machine (Bio-Rad).

### Flow cytometry.

Cell collection and processing were performed as described previously (40). The superior right lung lobe was mashed through a cell strainer and treated with ACK lysis buffer (10-5483; Lonza). Cells were pelleted, washed with 1% BSA–0.2 mM EDTA in PBS, and incubated with Live/Dead fixable aqua fluorescent reactive dye (L34966; Invitrogen), anti-mouse CD16/CD32 (Fc block, clone 2.4G2; 70-0161-V100; Tondo Biosciences), anti-mouse CD3e fluorescein isothiocyanate (clone 145-2011, 11-0031-85; eBioscience), anti-mouse CD4 PE/Cy7 (clone GK1.5, 100422; BioLegend), anti-mouse CD8a allophycocyanin/H7 (clone 53-6.7, 560182; BD Biosciences), and Ly6G-BV605 (clone RB6-8C5; BD). Cells were acquired on a BD Fortessa flow cytometer and gated on live CD3^+^ lymphocytes. Data were analyzed on FlowJo v10.1.

### Statistical analysis.

*In vitro* and *in vivo* analyses were performed in Prism 8 as described in the figure legends (GraphPad Software).

### Clinical cohort.

*IFI44* and *IFI44L* expression was analyzed in a published clinical cohort of febrile infants with either moderate or severe RSV infection (13). The microarray gene expression data set (GSE72810) was retrieved from the National Institutes of Health Gene Expression Omnibus database (41) using the GEOquery package (42) in R (43). Normalization was performed using robust spline normalization (RSN) from the lumi package (44), followed by a log transformation. Patients with suspected or confirmed bacterial infection were removed (*n* = 4). Cohort demographics are described in the associated figures. Prior to differential expression, probes were removed if the expression was not above 6 in at least 4 samples. Differential expression was performed using Limma, and expression values were normalized using robust spline normalization and a log transformation; plotted *P* values were not adjusted for multiple testing (values in the table in Fig. 7 are Benjamini-Hochberg corrected). Interferon-stimulated genes were downloaded from KEGG.

### Ethics.

All animal experiments were maintained in accordance with UK Home Office regulations, the UK Animals (Scientific Procedures) Act 1986, and reviewed by an Animal Welfare and Ethical Review Body. The work was done under PPL P4EE85DED. Clinical data presented were collected in a previous study (13). Written informed consent was obtained from parents or guardians using locally approved research ethics committee permissions (St Mary’s Research Ethics Committee [REC 09/H0712/58 and EC3263]; Ethical Committee of Clinical Investigation of Galicia [CEIC ref 2010/015]; UCSD Human Research Protection Program no. 140220; and Academic Medical Centre, University of Amsterdam [NL41846.018.12 and NL34230.018.10]).
